# Effects of Summer Heat Stress on Physiological Parameters and Reproductive Efficiency of Barbarine Ewes Under Natural Outdoor or Indoor Conditions

**DOI:** 10.1002/jez.70101

**Published:** 2026-06-02

**Authors:** Samia Khnissi, Dorra Aouadi, Hela Chalouati, Mourad Rekik, Imène Ben Salem

**Affiliations:** ^1^ Laboratory of Animal and Forage Production, National Institute of Agronomic Research of Tunisia University of Carthage Tunis Tunisia; ^2^ International Center for Agricultural Research in the Dry Areas (ICARDA) Tunis Tunisia; ^3^ Department of Animal Production, Service of Zootechnics and Agricultural Economy, National School of Veterinary Medicine Sidi Thabet University of Manouba La Manouba Tunisia

**Keywords:** Barbarine ewes, heat stress, physiological responses, reproductive performance, summer breeding, THI

## Abstract

Climate change and rising ambient temperatures increasingly challenge sheep production systems in arid and semi‐arid regions, particularly during the summer breeding season. This study evaluated the effects of heat stress and direct solar exposure on physiological responses, metabolic profile, and reproductive performance of Barbarine ewes, a fat‐tailed breed adapted to harsh environments. A total of 112 non‐lactating multiparous ewes were allocated to two groups during summer mating: an indoor group protected from solar radiation (IDGp; *n* = 58) and an outdoor group exposed to direct sunlight (ODGp; *n* = 54). Both groups experienced severe to extreme heat stress for more than 65% of the experimental period. Sun exposure significantly increased rectal temperature (+0.2 to +0.6°C), respiratory rate (up to 130 vs 61 breaths/min), and heart rate (up to 95 vs 87 beats/min) in outdoor ewes (ODGp) compared to indoor ewes (IDGp), indicating a higher physiological heat load in sun‐exposed animals. The temperature–humidity index (THI) remained comparable between groups (≈24.7), suggesting comparable THI values. Metabolic responses were characterized by increased plasma concentrations of triglycerides (+36%), total proteins (+7.5%), urea (+13%), creatinine (+34%), and potassium (+5%), along with a decrease in cholesterol levels (−15%) in ODGp compared to IDGp. Despite these physiological and metabolic adjustments, body condition score and live weight were maintained in both groups. Reproductive performance was not affected by solar exposure, with estrus occurrence exceeding 94%, an ovulation rate of 1.17, and no significant differences in fertility (79.3% vs 77.8%), prolificacy, or fecundity between groups. These findings indicate that, although direct solar radiation increases physiological strain, it does not impair reproductive function under comparable ambient thermal conditions. Barbarine ewes thus exhibit strong functional resilience to heat stress, highlighting their suitability for extensive production systems in hot and dry environments.

## Summary

Barbarine ewes demonstrate strong resilience to summer heat stress, supporting their suitability for extensive systems in hot and dry climates.

## 1 Introduction

Sheep husbandry plays a crucial role in sustaining the livelihoods of large populations, especially in hot, arid, and semi‐arid climates (Sejian et al. 2017). Climate change has significantly impacted sheep production, particularly in extensively managed systems, where sheep are raised outdoors and are directly exposed to environmental stressors such as temperature, relative humidity, and solar radiation (Feleke et al. 2016; Rojas‐Downing et al. 2017; Castillo et al. 2021; Mohamed‐Brahmi et al. 2024). These factors often result in heat stress (HS), which can have both direct and indirect effects on livestock productivity (Al‐Dawood 2016; Sejian et al. 2019; Castillo et al. 2021).

Despite these challenges, indigenous sheep breeds from hot, arid, and semi‐arid regions tend to be more resilient to climatic variations, adapting better to harsh environmental conditions and resource scarcity (Sejian et al. 2017; De Barbieri et al. 2023). However, the extent to which these breeds can withstand rapid temperature increases, reduced precipitation, and more frequent extreme weather events remains uncertain (Kumar et al. 2017).

When exposed to HS, sheep employ thermoregulatory mechanisms such as increased respiration and blood flow redistribution toward the periphery to dissipate excess body heat and regulate their core temperature (Macías‐Cruz et al. 2018; Sejian et al. 2018). However, when these mechanisms fail to adequately disperse the heat load, rectal temperatures rise, leading to a cascade of physiological changes. One of the primary consequences of HS in sheep is a reduction in feed intake, along with increased water consumption and disruptions in hormonal secretions and reproductive functions (Marai et al. 2008; Rathwa et al. 2017; Tüfekci 2023; Ben Moula et al. 2024). The reproductive efficiency of ewes may be compromised, with adverse effects on mating behavior, fertility, embryo development, and increased embryonic and perinatal lamb mortality (van Wettere et al. 2021; Robertson and Friend 2022; Ngcobo et al. 2025).

In Tunisia, sheep are a key component of the agricultural economy, with around 6.5 million animals contributing significantly to the livelihoods of farmers (FAO 2022). The Barbarine, a native fat‐tailed breed, accounts for nearly 50% of the sheep population (OEP 2020). Despite its prominence, Barbarine sheep are raised under diverse extensive and agro‐pastoral systems, often relying on low‐input management practices under harsh environmental conditions (Ibidhi et al. 2018; Mohamed‐Brahmi et al. 2024).

Research shows that the Barbarine breed has developed mechanisms to cope with harsh environmental conditions (Atti et al. 2004; Rekik et al. 2005; Lassoued et al. 2008; Ben Salem et al. 2011; Mleil et al. 2011). However, while these adaptations help ensure survival, they may come at the cost of reproductive performance. Many flocks are managed with summer mating practices, as the sexual season for local breeds typically begins in late July, aligning with a decreasing photoperiod (Khaldi 1984; Lassoued and Khaldi 1995). This period also coincides with the peak summer temperatures, which begin to rise in March, peak in July, and remain high until September.

In Tunisia, climate projections indicate a temperature rise of 0.7°C to 2.6°C and a decrease in precipitation of 4.1% to 6.7% by 2050 (USAID 2018). As climate change is expected to increase the duration and intensity of the warm period, these environmental stressors may have a greater impact on the reproductive performance of Barbarine ewes.

While the effects of heat stress on sheep physiology and reproduction are well documented, the specific contribution of direct solar radiation under comparable thermal load conditions remains poorly characterized. In extensive systems, animals are simultaneously exposed to high ambient temperatures and intense solar radiation, yet their combined effects are rarely distinguished. This gap limits our understanding of whether solar exposure exacerbates physiological stress to a level that may compromise reproductive performance.

Therefore, this study aimed to evaluate the impact of direct solar exposure on physiological responses, metabolic profile, and reproductive performance in Barbarine ewes under summer heat stress conditions by comparing indoor and outdoor management systems.

## 2 Material and Methods

### 2.1 Study Site and Climatic Conditions

This study was conducted at the Experimental Unit of the Institut National de la Recherche Agronomique de Tunisie, located at Bou Rébiâa (latitude 36°38′ N and longitude 10°17′E). The climate is of Mediterranean type, characterized by irregular rainfall and a long dry season. The highest and the lowest temperatures recorded during summer and winter seasons are 35.6°C and 4.4°C, respectively, and the annual average rainfall is 440 mm.

Environmental conditions, including ambient temperature (T) and relative humidity (RH), were monitored throughout the experimental period. Temperature was recorded both inside and outside the sheepfold using digital max–min thermometers. Relative humidity was measured inside the sheepfold using a digital hygrometer, while outdoor values were obtained from available climatic databases of the National Institute of Meteorology (INM), Tunis, Tunisia.

All measuring devices were placed inside protective white plastic shields and positioned at approximately 1 m above ground level to avoid direct animal interference.

The temperature–humidity index (THI) was calculated to assess the level of heat stress using the equation proposed by Marai et al. 2007: THI = T − (0.31 − 0.31 × RH) × (T − 14.4), where T is the ambient temperature (°C) and RH is the relative humidity (%).

**Figure 1:**
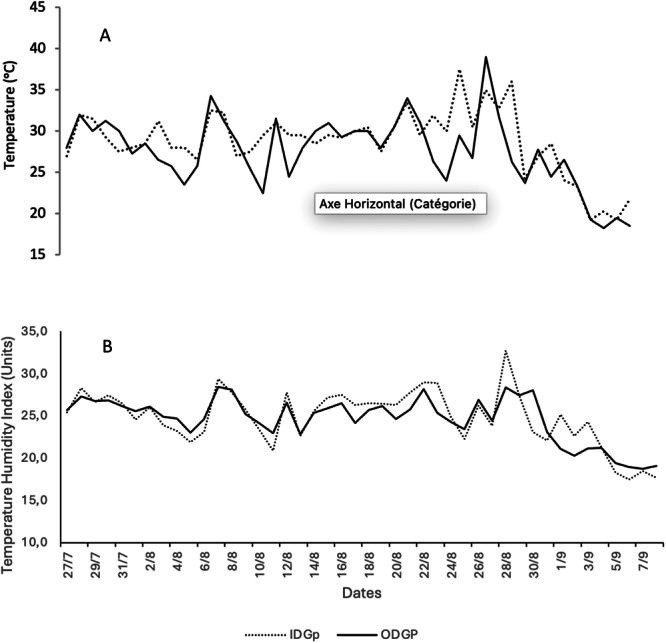
Daily variation of ambient temperature (A) and temperature–humidity index (THI) (B) during the experimental period in indoor (IDGp) and outdoor (ODGp) groups of Barbarine ewes.

Climatic conditions recorded during 44 days of experimental periods for indoor and outdoor groups are presented in Table 1 and Figure 1. The temperature average was 28.8 ± 3.98°C and 27.6 ± 4.29°C, respectively, for INGp and ODGp (Table 1). The average minimum temperature in IDGp was 19.4°C and the maximum 35.5°C; in ODGp, the minimum was 17.9°C and the maximum 39.9°C (Figure 1). RH and THI averages for IDGp were 33.9% and 24.8 units, respectively. Figures for ODGp were 62.1% and 24.8 units, respectively, for RH and THI (Table 1 and Figure 1).

**Table 1:** Climatic conditions recorded during the experimental period in indoor (IDGp) and outdoor (ODGp) groups of Barbarine ewes.

Item	Average (±SE)	Maximum	Minimum
Indoor group (IDGp)			
Temperature (°C)	28.8 ± 3.98	35.5	19.6
Relative humidity (%)	33.9 ± 10.40	59.6	14.1
Temperature‐humidity index (units)	24.8 ± 2.61	32.7	17.5
Outdoor group (ODGp)			
Temperature (°C)	27.6 ± 4.29	39.9	17.6
Relative humidity (%)	62.1 ± 3.60	69	57
Temperature–humidity index (units)	24.6 ± 2.11	28.4	18.7

Abbreviation: SE, standard error.

### 2.2 Animals and Management

The experiment was performed from July 24 to September 15. After this date, a follow‐up of the pregnant ewes and lambing was carried out. A total of 112 non‐lactating multiparous adult ewes of the Barbarine fat tail breed were selected for the experiment. Animals were allocated to two groups balanced for LW and age. The first group (IDGp) was housed in a sheepfold with a built roof and walls and a solid floor with straw as bedding material, and with natural ventilation. Animals were maintained indoors at prevailing ambient temperatures and away from the sun (*n* = 58; LW = 43.5 ± 5.9 kg; age = 4.1 ± 1.8). The second group (ODGp) was placed outdoors in a large, fenced area and exposed directly to solar radiation (*n* = 54; LW = 43 ± 5.4 kg; age = 4.1 ± 1.9). After allocation to each group, rams (previously separated from ewes for a period of 1 month), were put in contact with the females on July 24.

Animals in both groups were managed under similar feeding conditions throughout the experimental period. All ewes were fed 0.8 kg/day of oat hay and 0.5 kg/day of barley, which covered the maintenance requirements of mature ewes according to Jarrige (1988) feeding standards. Oat hay was offered twice daily, in the morning (08:00 h) and in the afternoon (17:00 h), while barley was provided as a single meal in the morning (09:00 h). Fresh water was available ad libitum throughout the experimental period.

Feed intake was not individually measured. However, both groups received the same diet under the same feeding schedule, and no intentional differences in feeding management were introduced between groups. In addition, outdoor ewes were maintained in a large, fenced area without access to grazing, ensuring that feed intake originated exclusively from the provided ration. This management approach was implemented to minimize variability in feed intake between groups and to ensure that any observed differences were primarily attributable to housing conditions rather than nutritional factors.

### 2.3 Treatments and Measurements

#### 2.3.1 Physiological Parameters

Rectal temperature (RT) was recorded weekly at 12.00 h each time with a digital thermometer introduced rectally for a minute. Respiratory rate (RR) and heart rate (HR) were recorded three times during the experiment (Days 3, 15, and 34) on a subset of 25 ewes per group, randomly selected from the experimental population. This sampling strategy was adopted due to the labor‐intensive and time‐consuming nature of these measurements, as well as to limit animal handling stress. The selected animals were considered representative of their respective groups in terms of age, physiological status, and body condition.

RR was recorded by observing the flank movements of the ewes for 1 min. HR was measured using a stethoscope placed over the heart area, and the number of beats per minute was recorded. All measurements were performed at 13:00 h in each group.

LW was determined every 2 weeks using a scale with a precision of 0.1 kg. Body Condition Score (BCS) was simultaneously measured using the scale (1–5) proposed by Russel et al. 1969 for dorsal BCS and the scale proposed by Atti (1991) for caudal BCS.

### 2.4 Blood Sampling and Metabolic Parameters Analysis

Blood samples were individually collected from the jugular vein every week in the morning before watering and feeding, to determine plasma concentrations of glucose (GLU), urea, creatinine (CRE), total protein (TP), cholesterol (CHOL), triglyceride (TRI), sodium (Na), potassium (K), calcium (Ca), and phosphorus (Pho). Blood samples were collected in 10‐mL vacuum tubes and centrifuged for 15 min at 3500 rpm. After blood plasma recovery, metabolites and electrolytes were determined using electrolyte analyzer Genuis (Model GE200B; N.C: 300401101712; Genuis, MA, USA).

For thyroxine (T4) assessment, blood was taken on two periods at day 6 and day 38 of the experiment. Each time blood was collected from 10 ewes in each group every 4 h for 24 h (2 p.m., 6 p.m., 10 p.m., 02 a.m., 06 a.m., and 10 a.m.). Samples were centrifuged at 3500 × g for 15 min to separate the plasma, which was deposited and stored in 1.8‐mL vials by duplicate at −20°C until thyroxine analysis. The plasma thyroxine was performed through a radioimmunoassay (Immunotech, Beckman Coulter, Brea, California, USA) according to the manufacturer's instructions. The technique had a sensitivity of 2.34 pM/L. The coefficients of variation were below or equal to 10.29% and below or equal to 7.58%, respectively, for intra‐ and inter‐assay.

### 2.5 Reproductive Parameters

The estrous behavior in ewes was determined using rams fitted with an apron, while ovulation was determined by endoscopy as indicated by Thimonier et al. (1969). The heat control was done throughout the day. Once detected in estrus, the ewe was presented to the ram for breeding. At parturition, lambing date and number of lambs born were recorded individually for each ewe. Response to estrus (percentage of ewes in estrus), length of estrus cycle (time interval between an estrous and the subsequent estrous), ovulation rate (number of CL per number of ewes in estrus), fertility (percentage of ewes lambed from ewes mated), prolificacy (percentage of lambs born from ewes lambed), fecundity (percentage of lambs born from ewes mated), abortion rate (percentage of ewes aborting from ewes mated) were determined from collected information.

### 2.6 Statistical Analysis

Live weight, dorsal and caudal BCS, RT, RR, HR, metabolites (glucose, urea, creatinine, total protein, cholesterol, triglyceride, sodium, potassium, calcium, and phosphorus), length of estrous cycle, and ovulation rate were analyzed by analysis of variance of SAS (ANOVA), considering sun exposure or housing indoors as the treatment. Mean comparisons were performed with the option PDIFF when significant differences were detected at *α* = 0.05 (SAS Institute Inc 2017). Plasma thyroxine concentrations were analyzed with the same design but using repeated measures over time; the model included effects of sun exposure, time (hours of the sampling), and the interaction between those main factors. All results were expressed as least‐squares means (LS means) ± SEM (Standard Error of Mean), and statistical significance was set at *p* < 0.05.

Variables expressed in percentages (distribution of temperature–humidity index (THI) classes in both groups, occurrence of heat stress classes, ewes ovulating, ewes in estrus, fertility, prolificacy, fecundity, and abortion) were analyzed with the chi‐square test. Statistical significance was set at *p* < 0.05.

## 3 Results

### 3.1 Climatic Conditions

**Figure 2:**
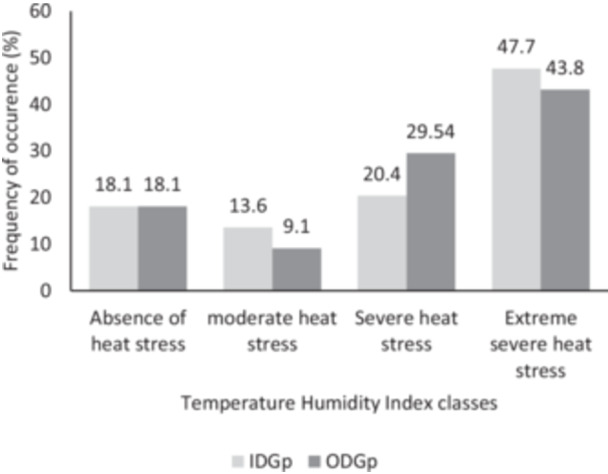
Percentage distribution of days (%) within each temperature–humidity index (THI) class over the entire experimental period in indoor (IDGp) and outdoor (ODGp) groups of Barbarine ewes. THI classes were defined according to Marai et al. (2007).

Chi‐square (*χ* ^2^) analysis revealed no significant difference between the two groups in the distribution of temperature–humidity index (THI) classes between indoor (IDGp) and outdoor (ODGp) groups (*p* > 0.05; Figure 2). According to the classification of Marai et al. (2007), in both groups, most of the experimental period was characterized by severe to extremely severe heat stress conditions, accounting for 68% and 73% of the total number of days in IDGp and ODGp, respectively.

### 3.2 Live Weight and Body Condition Score

**Figure 3:**
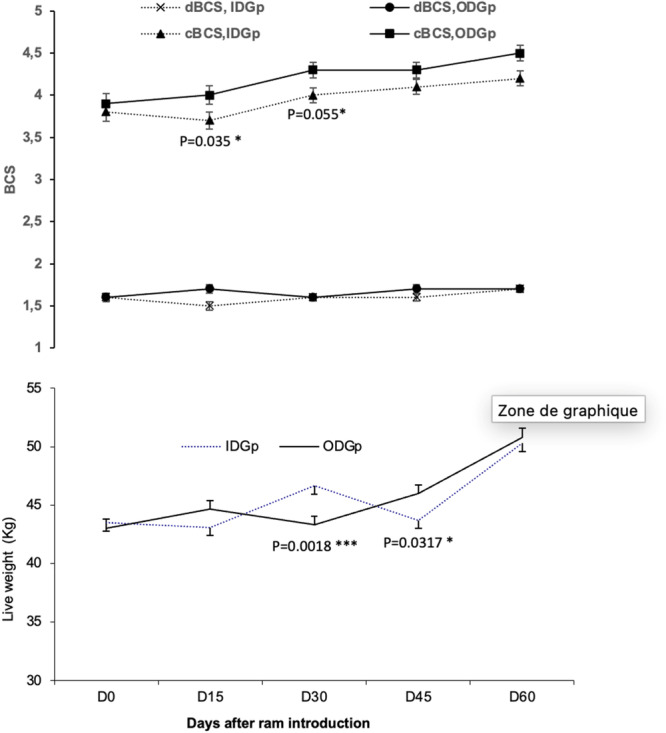
Changes body condition score (BCS) (A) and live weight (kg) (B) of Barbarine ewes in indoor (IDGp) and outdoor (ODGp) groups during the experimental period. Values are expressed as LS means ± SEM. * *p* < 0.05; *** *p* < 0.001.

Live weight showed moderate variations over time in both groups (Figure 3B). Although some differences were observed at specific time points, no consistent effect of housing conditions was detected throughout the experimental period. Overall, LW increased slightly in both groups from the beginning to the end of the experiment, reaching 50.33 kg and 50.78 kg in IDGp and ODGp, respectively.

Similarly, body condition score (BCS), both dorsal and caudal, remained relatively stable over time in both groups (Figure 3A). No clear effect of sun exposure was observed, except for a slightly higher caudal BCS in outdoor ewes at days 15 and 30.

### 3.3 Physiological Parameters

RT was higher in the ODGp than in the IDGp for the measurements of week 1, 2, and 5 (P < 0.05, Table 2). On average, ewes had 0.6°C, 0.2°C, and 0.2°C higher rectal temperatures in ODGp, respectively, in weeks 1, 2, and 5. Mean weekly RR were significantly higher (*P* < 0.05) in ODGp than IDGp (Table 2). The extent of variation was from a minimal value of 38.7°C for both groups to a maximum value of 40.0°C and 40.9°C, respectively, for IDGp and ODGp.

**Table 2:** Weekly physiological parameters (rectal temperature, respiratory rate, and heart rate) were measured in indoor (IDGp) and outdoor (ODGp) groups of Barbarine ewes during the experimental period.

Physiological parameters	IDGp	ODGp	*P*‐value
Temperature (°C)			
Week 1	39.2 ± 0.04	39.8 ± 0.04	0.0001
Week 2	39.2 ± 0.04	39.4 ± 0.05	0.0003
Week 3	39.4 ± 0.03	39.4 ± 0.03	0.9581
Week 4	39.3 ± 0.03	39.3 ± 0.03	0.8988
Week 5	39.6 ± 0.04	39.8 ± 0.04	0.0277
Week 6	39.3 ± 0.03	39.3 ± 0.03	0.9129
Number of observations	58	54	
Respiratory rate (breaths/min)			
Week 1	44 ± 2.5	70 ± 2.6	0.0001
Week 2	53 ± 4.2	100 ± 4.4	0.0001
Week 3	61 ± 2.4	130 ± 2.5	0.0001
Number of observations	25	25	
Heart rate (beats/min)			
Week 1	77 ± 2.03	90 ± 2.12	0.0001
Week 2	86 ± 2.44	93 ± 2.54	0.039
Week 3	87 ± 1.14	95 ± 1.18	0.0001
Number of observations	25	25	

*Note:* Values are expressed as lsmean ± SEM.

Differences were considered significant at *p* < 0.05.

Abbreviations: IDGp, indoor group; ODGp, outdoor group.

As for RR, values varied from 44 ± 3 to 61 ± 2 breath/min and from 70 ± 3 to 130 ± 2.5 breath/min, respectively for IDGp and ODGp (Table 2). Ewe exposed to sun showed higher HR than those housed indoor (*p* < 0.05, Table 2). Recorded values ranged from 77 ± 2 to 87 ± 1 beats/min and from 90 ± 2 to 95 ± 2 beats/min, respectively for IDGp and ODGp.

### 3.4 Blood Metabolic Parameters

Statistical analysis showed significant metabolic changes characterized by increased values of TRI, TP, CRE, urea, and K in the ODGp in comparison to the IDGp. CHOL was lower in ODGp compared to IDGp. Blood metabolic concentrations are reported in Table 3.

**Table 3:** Blood metabolite concentrations (LS mean ± SEM) in outdoor (ODGp) and indoor (IDGp) groups of Barbarine ewes during the experimental period.

Parameters	LS means±SEM		*P*‐Value
	ODGp	IDGp	
Glucose (mmol/L)	3.08 ± 0.04	3.03 ± 0.04	0.400
Cholesterol (mmol/L)	1.84 ± 0.04	2.17 ± 0.04	0.040
Triglycerides (mmol/L)	0.34 ± 0.01	0.25 ± 0.01	0.010
Total proteins (g/L)	77.08 ± 0.83	71.70 ± 0.81	0.008
Creatinine (µmol/L)	71.6 ± 2.94	53.50 ± 2.87	0.008
Urea (mmol/L)	3.87 ± 0.07	3.43 ± 0.07	0.007
Na^+^ (mmol/L)	141.53 ± 0.46	141.90 ± 0.44	0.457
K^+^ (mmol/L)	4.30 ± 0.04	4.08 ± 0.04	0.015
Ca^++^ (mmol/L)	1.78 ± 0.05	1.77 ± 0.05	0.115
Phosphorus (mmol/L)	1.46 ± 0.03	1.46 ± 0.03	0.362

*Note:* Values are expressed as lsmean ± SEM (standard error of the mean). Differences were considered significant at *p* < 0.05.

Abbreviations: IDGp, indoor group; ODGp, outdoor group.

**Figure 4:**
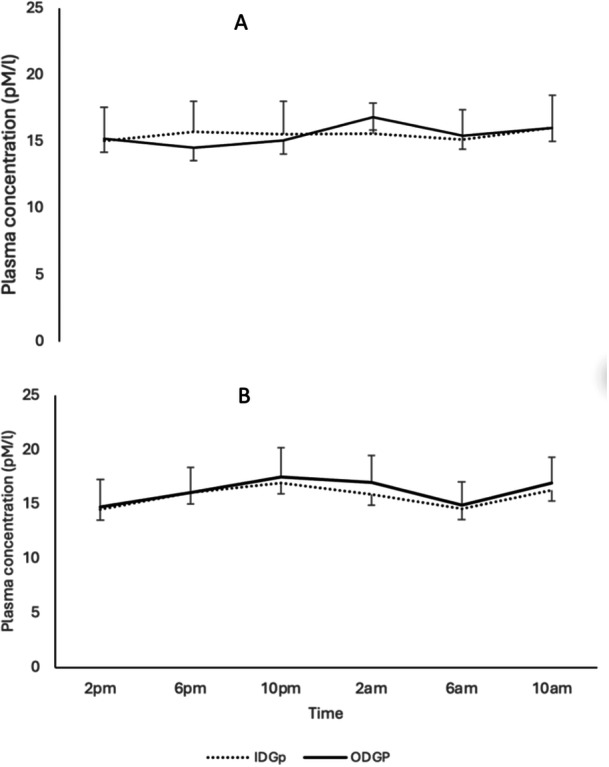
Plasma thyroxine (T4) concentration (pM/L) measured over a 24‐h period in indoor (IDGp) and outdoor (ODGp) groups of Barbarine ewes at (A) 6–7 days and (B) 37–38 days after ram introduction. Values are expressed as LS mean ± SEM.

Results of thyroxin levels during two sampling periods (i) end of July, i.e., day 6 to day 7 of the experiment, and (ii) end of August, i.e., day 37 to day 38 of the experiment are presented in Figure 4. No significant effect of housing (indoor vs outdoor) was observed on plasma thyroxine concentrations. Mean plasma thyroxine (T4) concentration was 15.33 ± 2.69 pmol/L in sun‐exposed ewes and 15.35 ± 2.67 pmol/L in indoor‐housed ewes.

Regardless of housing conditions, a significant effect of sampling time on plasma T4 concentrations was observed during the August sampling period (*p* < 0.01), with values ranging from 14.95 ± 2.59 pM/L in the afternoon (02:00 p.m.) to 17.83 ± 2.50 pM/L at night (10:00 p.m.), indicating a clear diurnal variation.

### 3.5 Reproductive Parameters

As presented in Table 4, non‐significant differences were observed between IDGp and ODGp regarding the occurrence of estrus, ovulation rate, fertility prolificacy, fecundity, and abortion percentage. Fifty‐five ewes out of 58 and 51 out of 54 ewes presented estrus signs, respectively in IDGp and ODGp.

**Table 4:** Reproductive performance parameters in indoor (IDGp) and outdoor (ODGp) groups of Barbarine ewes.

Parameters	IDGp	ODGp	*P*‐value
Ewes in estrus* (%)	94.8 (55/58)	94.4 (51/54)	0.900
Ewes with one estrus (%)	78.2 (43/55)	80.4 (41/51)	0.450
Ewes with two estrus (%)	21.8 (12/55)	19.6 (10/51)	0.450
Length of the estrus cycle (days, ±SEM)	18.37 ± 2.39	17.87 ± 2.64	0.700
Ewes ovulating (%)	96.15 (54/55)	100 (51/51)	0.900
Single ovulation (%)	83.3 (45/54)	80.4 (41/51)	0.250
Double ovulation (%)	16.6 (9/54)	19.6 (10/51)	0.250
Ovulation rate	1.16	1.19	0.600
Fertility (%)	79.3 (46/58)	77.8 (42/54)	0.100
Prolificacy (%)	104.3 (48/46)	109.5 (46/42)	NS
Fecundity (%)	82.7 (48/58)	85.18 (46/54)	NS
Abortion (%)	6.9 (4/58)	5.55 (3/54)	NS

*Note: P*‐values indicate differences between groups. Differences were considered significant at *p* < 0.05.

Abbreviations: IDGp, indoor group; ODGp, outdoor group; SEM, standard error of mean.

* Estrus behavior was monitored for up to 24 days after ram introduction.

Reproductive parameters were not affected by sun exposure (*p* > 0.05). Corresponding values for fertility, prolificacy, and fecundity were 79.3%, 104.3, and 82.7 for IDGp, and 77.8%, 109.5, and 85.18 in ODGp (Table 4).

Assessment of potential reproductive loss based on the total number of lambs born and total number of corpora lutea observed by endoscopy was 33.4 and 35.2% for INGp and ODGp, respectively. If we take into consideration females having aborted, the real fertility rate reached 86.2% and 83.3% respectively in IDGp and ODGp (Table 4). The proportion of ewes that failed to conceive at the end of the mating period was 13.8% and 16.7% for INGp and ODGp, respectively.

## 4 Discussion

### 4.1 Climatic Conditions

During summer, climatic conditions recorded in the present study clearly reflected heat stress (HS), as average temperatures in IDGp and ODGp (27.6°C and 28.8°C, respectively) exceeded the upper limit of the thermoneutral zone generally reported for sheep, although this threshold may vary depending on breed and environmental conditions (Marai et al. 2007; Sejian et al. 2018). During the experimental period, ewes in IDGp experienced 25 days of heat stress out of 44, compared to 33 days in ODGp.

The temperature–humidity index (THI) further confirmed these conditions, with mean values (24.8 ± 2.60 in ODGp and 24.6 ± 2.11 in IDGp) corresponding to severe heat stress (Marai et al. 2007). In addition, the frequency of THI classes did not differ significantly between indoor and outdoor groups, suggesting that indoor housing may have provided only limited mitigation of the ambient thermal load.

These findings indicate that natural ventilation alone was insufficient to reduce heat load within the sheepfold under hot environmental conditions. Warm outdoor air entering the building may limit the cooling capacity of naturally ventilated systems (Papanastasiou et al. 2018).

Improving thermal conditions in such systems may require additional management strategies, including enhanced ventilation, insulation, and shading, as well as appropriate nutritional adjustments to support animal resilience under heat stress (Papanastasiou et al. 2014; Todaro et al. 2015).

### 4.2 Live Weight and Body Condition Score

Despite exposure to severe summer heat stress, Barbarine ewes maintained overall body condition and live weight throughout the experimental period. Although transient differences in live weight were observed between groups, no consistent effect of housing conditions was detected. Both indoor and outdoor ewes exhibited a gradual increase in live weight, indicating that heat stress did not lead to net body weight loss.

Body condition score (BCS) remained relatively stable throughout the experimental period. Although some differences were observed at specific time points, particularly for caudal BCS during the early phase of the experiment, these differences were not maintained over time. As BCS is closely related to body energy reserves (Atti et al. 2004; Rekik et al. 2005; Ben Salem et al. 2011; Moradi et al. 2012), its stability suggests that animals were able to preserve their energy balance despite prolonged exposure to high temperatures. Similar responses have been reported in other sheep breeds under heat stress conditions (Marai et al. 2007; Chay‐Canul et al. 2011).

The fluctuations observed in live weight likely reflect short‐term adjustments in feed intake, digestibility, and metabolic activity rather than a direct effect of sun exposure (Marai et al. 2007; Arero and Ozmen 2025).

The ability of Barbarine ewes to preserve both live weight and body condition under such conditions highlights their adaptive capacity. Morphological and physiological traits, including fat‐tail deposition and the capacity to store body reserves, may contribute to maintaining energy balance during periods of environmental stress (Atti et al. 2004; Pourlis 2011; Chedid et al. 2014). These adaptive mechanisms are characteristic of fat‐tailed breeds and contribute to their resilience in arid and semi‐arid environments (Chay‐Canul et al. 2011; Moradi et al. 2012).

Although animals were managed under similar feeding conditions and outdoor ewes had no access to grazing, individual feed intake was not measured. Therefore, potential variations in intake between animals cannot be excluded.

### 4.3 Physiological Parameters

Although thermal conditions were not experimentally controlled, the similar THI values observed between indoor and outdoor groups suggest that animals were exposed to comparable overall heat load. In this context, the differences observed in physiological responses may be partly attributed to the additional effect of direct solar radiation. Accordingly, exposure to heat stress in the present study was associated with increases in rectal temperature, respiratory rate, and heart rate, which are recognized as key physiological indicators of thermoregulatory response in sheep (Marai et al. 2007; McManus et al. 2009; Al‐Dawood 2016). Such responses are commonly reported under heat stress conditions, with respiratory rate potentially increasing two‐ to five‐fold and heart rate by 20% to 50% (Silanikove 2000; Marai et al. 2007; Alhidary et al. 2012). These changes reflect an increased metabolic heat load and activation of heat dissipation mechanisms, including panting and peripheral vasodilation (McManus et al. 2020; Santos et al. 2021; Vieira et al. 2021).

Rectal temperature values remained within the upper physiological range reported for sheep (38.3–39.9°C; Kadzere et al. 2002; Reece and Rowe 2017). Rectal temperature differed between groups at specific time points, with significantly higher values consistently observed in outdoor ewes during the first, second, and fifth weeks. These variations suggest dynamic thermoregulatory responses over time rather than a constant effect of housing conditions, reflecting the variable nature of physiological adaptation to heat stress (Sejian et al. 2018; Bernabucci et al. 2010).

Respiratory rate increased markedly in outdoor ewes compared to indoor ewes, reflecting a primary thermoregulatory response to heat stress. Increased respiration enhances evaporative heat loss and is considered one of the most effective mechanisms for dissipating excess body heat in small ruminants (Silanikove 2000; Santos et al. 2021; Vieira et al. 2021; Arero and Ozmen 2025). Heart rate was also higher in sun‐exposed ewes, likely reflecting increased peripheral blood flow to facilitate heat dissipation (Al‐Dawood 2016; McManus et al. 2020).

Overall, the magnitude of these responses suggests that Barbarine ewes effectively activate thermoregulatory mechanisms under heat stress, as evidenced by increased respiratory and cardiovascular activity in outdoor compared to indoor animals. This supports their known adaptive capacity to arid and semi‐arid environments, particularly in fat‐tailed breeds (Ben Salem et al. 2011; Rekik et al. 2005; McManus et al. 2009; Renaudeau et al. 2012).

### 4.4 Blood Metabolic Parameters

Heat stress is associated with alterations in metabolic pathways and physiological adaptations (Yuan et al. 2025). Under HS conditions, reduced feed intake and a shift in energy metabolism are commonly reported, leading to modifications in protein and nitrogen metabolism (Baumgard and Rhoads 2013; Sejian et al. 2018).

In the present study, the ODGp exhibited higher levels of triglycerides, total protein, creatinine, urea, and potassium compared to the IDGp. These changes can be interpreted as part of an integrated physiological response to heat stress, involving adjustments in protein, lipid, and electrolyte metabolism aimed at maintaining homeostasis (Silanikove 2000; Sejian et al. 2018). Although body weight and body condition score remained stable, indicating no major negative energy balance, the observed metabolic changes suggest a reorganization of nutrient utilization rather than a substantial mobilization of body reserves.

Increases in TP and urea have been associated with enhanced protein turnover and altered nitrogen balance, partly driven by increased maintenance energy requirements (Mehaba et al. 2019). The increase in triglyceride levels may reflect alterations in lipid metabolism, possibly related to changes in energy utilization (Čukić et al. 2023). In addition to this increase in triglycerides, plasma cholesterol levels were significantly reduced in sun‐exposed ewes, suggesting a differential regulation of lipid metabolism, possibly involving alterations in hepatic lipid metabolism or increased utilization for cellular functions and steroidogenesis (Silanikove 2000; Sejian et al. 2018; Čukić et al. 2023).

Elevated creatinine (CR) levels may reflect alterations in muscle metabolism and renal function, as well as potential hemoconcentration effects, although hydration status was not directly assessed in this study (Hamzaoui et al. 2013; Baumgard and Rhoads 2013; Čukić et al. 2023).

Although reduced feed intake is commonly reported under heat stress conditions, it was not measured in this study and therefore cannot be confirmed, but it may have contributed to the observed metabolic adjustments.

Plasma T4 levels are influenced by environmental conditions, nutrition, and physiological status. Previous studies have shown that thyroid activity is generally reduced under heat stress, indicating an adaptive reduction in metabolic rate and heat production (Todini 2007; Indu et al. 2015), which contributes to limiting endogenous heat production (Omidi et al. 2015; Rodrigues et al. 2023).

However, no significant differences in T4 concentrations were observed between indoor and outdoor ewes, despite differences in sun exposure. This suggests that both groups exhibited comparable endocrine adaptation to thermal stress. Similar findings have been reported in indigenous goat breeds, where no significant changes in T4 levels were observed under comparable thermal conditions (Prathap et al. 2017).

### 4.5 Reproductive Performance

The present study revealed that fertility rates (79.3% and 77.8% for ewes housed indoors and those exposed to direct solar radiation, respectively) obtained after mating under summer heat stress were lower than typical fertility rates observed during spring mating for the same breed (Khaldi and Farid 1981; Abdennebi 1990; Brahmi et al. 2011). This reduction in fertility is consistent with previous findings indicating that heat stress during the mating period can impair fertilization, embryo survival, and overall pregnancy rates (González‐Bulnes et al. 2011). Similar reductions in fertility during hot seasons have been reported in various sheep breeds, with values around 74% observed in Pelibuey ewes under tropical conditions (Fuentes and Chemineau 1989). In Merino flocks, each additional day with temperatures exceeding 32°C has been associated with a decrease in fertility of approximately 3.5%, highlighting the cumulative impact of prolonged heat exposure on reproductive performance (Kleemann and Walker 2005). However, the magnitude of these effects may vary depending on breed and adaptive capacity (Sejian et al. 2018; Berihulay et al. 2019; McManus et al. 2020).

Several studies in sheep and cattle have shown that heat stress can reduce both the incidence and duration of estrus (Rensis and Scaramuzzi 2003; Naqvi et al. 2004; Bernabucci et al. 2010; Sejian et al. 2012), although these effects depend on the timing, duration, and intensity of thermal stress. In contrast, Barbarine ewes in the present study maintained high estrus and ovulation rates (over 90%) and exhibited stable estrous cycle lengths in both indoor (IDGp) and outdoor (ODGp) groups. These results indicate no significant effect of direct solar exposure on estrus expression or cycle characteristics. In addition, the use of the ram effect proved highly effective, with more than 85% of females displaying estrus behavior within the first 20 days following ram introduction (Hawken and Martin 2012), which may have contributed to sustaining reproductive activity under heat stress conditions.

In agreement with our findings, (Macías‐Cruz et al. 2016) reported similar estrus and ovulation rates between summer (heat stress conditions) and autumn (thermoneutral conditions) in Pelibuey ewes. Comparable observations have also been reported in other heat‐tolerant breeds, such as Malpura ewes, in which estrous cycle length remained unaffected under heat stress (Sejian et al. 2012). also reported stable progesterone levels in Pelibuey ewes under thermal stress, supporting the idea of endocrine stability in adapted breeds.

These findings suggest that, although heat stress may impair fertility outcomes, key reproductive processes such as estrus expression and ovulation can be maintained in locally adapted breeds. Importantly, the present results extend this understanding by showing that such resilience is preserved even under direct solar radiation, highlighting the ability of Barbarine ewes to maintain reproductive function under combined thermal and radiative stress conditions.

## 5 Conclusion

This study shows that, although summer heat stress induces marked physiological and metabolic adjustments in Barbarine ewes, reproductive performance remains largely unaffected, even under direct solar exposure. These findings highlight the strong adaptive capacity of this breed and its suitability for extensive production systems in hot environments. The dissociation observed between physiological stress responses and reproductive function suggests that key reproductive processes are preserved despite increased thermal load. In addition, the results provide new insights into the specific contribution of solar radiation under comparable ambient thermal conditions. From a practical perspective, strategies aimed at reducing heat load may further improve animal welfare without compromising reproductive efficiency.

## Funding

The authors have nothing to report.

## Conflicts of Interest

The authors declare no conflicts of interest.

## Data Availability

The data that support the findings of this study are available from the corresponding author upon reasonable request.

## References

[jez70101-bib-0001] Abdennebi, L. 1990. Analyse des Performances Zootechniques de 10 Années d’élevage d'un Troupeau Ovin Prolifique de Race Barbarine. Institut National Agronomique, 78–89.

[jez70101-bib-0002] Al‐Dawood, A. 2016. “Towards Heat Stress Management in Small Ruminants – A Review.” Annals of Animal Science 17: 59–88. 10.1515/aoas-2016-0068.

[jez70101-bib-0003] Alhidary, I. A. , S. Shini , R. a M. Al Jassim , and J. B. Gaughan . 2012. “Physiological Responses of Australian Merino Wethers Exposed to High Heat Load.” Journal of Animal Science 90: 212–220. 10.2527/jas.2011-3972. 21841087

[jez70101-bib-0004] Arero, G. B. , and O. Ozmen . 2025. “Effects of Heat Stress on Reproduction and Gene Expression in Sheep.” Animal Reproduction 22: e20240067. 10.1590/1984-3143-AR2024-0067. 40123803 PMC11927936

[jez70101-bib-0005] Atti, N. 1991. “Relations Entre I’État Corporel Et Les Dépôts Adipeux Chez La Brebis Barbarine.” Options Méditerranéennes‐Série Séminaires 13: 31–34.

[jez70101-bib-0006] Atti, N. , F. Bocquier , and G. Khaldi . 2004. “Performance of the Fat‐Tailed Barbarine Sheepin Its Environment: Adaptive Capacity to Alternationof Underfeeding and Re‐Feeding Periods. A Review.” Animal Research 53: 165–176. 10.1051/animres:2004012.

[jez70101-bib-0007] De Barbieri, I. , E. Navajas , F. Douhard , J. Conington , Z. Ramos , and G. Ciappesoni . 2023. “PL‐8 A Review of Sheep Resilience.” Animal ‐ Science Proceedings 14: 11–12. 10.1016/j.anscip.2023.01.009.

[jez70101-bib-0008] Baumgard, L. H. , and R. P. Rhoads . 2013. “Effects of Heat Stress on Postabsorptive Metabolism and Energetics.” Annual Review of Animal Biosciences 1: 311–337. 10.1146/annurev-animal-031412-103644. 25387022

[jez70101-bib-0009] Berihulay, H. , A. Abied , X. He , L. Jiang , and Y. Ma . 2019. “Adaptation Mechanisms of Small Ruminants to Environmental Heat Stress.” Animals 9: 75. 10.3390/ani9030075. 30823364 PMC6466405

[jez70101-bib-0010] Bernabucci, U. , N. Lacetera , L. H. Baumgard , R. P. Rhoads , B. Ronchi , and A. Nardone . 2010. “Metabolic and Hormonal Acclimation to Heat Stress in Domesticated Ruminants.” Animal 4: 1167–1183. 10.1017/S175173111000090X. 22444615

[jez70101-bib-0011] Brahmi, A. , M. A. Bouallègue , H. Bouzaiène , and G. Khaldi , 2011. Analyse de la Durabilité de l’élevage de la Race Barbarine Élevée sous des Conditions Tunisiennes du Système de Production Semi‐aride.

[jez70101-bib-0012] Castillo, D. A. , J. J. Gaitán , and E. S. Villagra . 2021. “Direct and Indirect Effects of Climate and Vegetation on Sheep Production Across Patagonian Rangelands (Argentina).” Ecological Indicators 124: 107417. 10.1016/j.ecolind.2021.107417.

[jez70101-bib-0013] Chay‐Canul, A. J. , A. J. Ayala‐Burgos , J. C. Ku‐Vera , J. G. Magaña‐Monforte , and L. O. Tedeschi . 2011. “The Effects of Metabolizable Energy Intake on Body Fat Depots of Adult Pelibuey Ewes Fed Roughage Diets Under Tropical Conditions.” Tropical Animal Health and Production 43: 929–936. 10.1007/s11250-011-9785-5. 21240653

[jez70101-bib-0014] Chedid, M. , L. S. Jaber , S. Giger‐Reverdin , C. Duvaux‐Ponter , and S. K. Hamadeh . 2014. “Review: Water Stress in Sheep Raised Under Arid Conditions.” Canadian Journal of Animal Science 94: 243–257. 10.4141/cjas2013-188.

[jez70101-bib-0015] FAO . 2022. FAOSTAT: Livestock Primary – Sheep. Food and Agriculture Organization of the United Nations. https://www.fao.org/faostat/en/#data/QCL.

[jez70101-bib-0016] Feleke, F. B. , M. Berhe , G. Gebru , and D. Hoag . 2016. “Determinants of Adaptation Choices to Climate Change by Sheep and Goat Farmers in Northern Ethiopia: The Case of Southern and Central Tigray, Ethiopia.” SpringerPlus 5: 1692. 10.1186/s40064-016-3042-3. 27752459 PMC5045456

[jez70101-bib-0017] Fuentes, J. L. , and P. Chemineau . 1989. “Fertilité de Brebis Pelibuey et Suffolk en Climat Tropical.” Annales De Zootechnie 38: 5–9. 10.1051/animres:19890102.

[jez70101-bib-0018] González‐Bulnes, A. , C. A. Meza‐Herrera , M. Rekik , H. Ben Salem , and R. T. Kridli . 2011. “Limiting Factors and Strategies for Improving Reproductive Outputs of Small Ruminants Reared in Semi‐arid environments.” In Semi‐Arid Environments: Agriculture, Water Supply and Vegetation, edited by K. M. Degenovine , 41–60. Nova Science Publishers Inc.

[jez70101-bib-0019] Hamzaoui, S. , A. A. K. Salama , E. Albanell , X. Such , and G. Caja . 2013. “Physiological Responses and Lactational Performances of Late‐Lactation Dairy Goats Under Heat Stress Conditions.” Journal of Dairy Science 96: 6355–6365. 10.3168/jds.2013-6665. 23958010

[jez70101-bib-0020] Hawken, P. a R. , and G. B. Martin . 2012. “Sociosexual Stimuli and Gonadotropin‐Releasing Hormone/Luteinizing Hormone Secretion in Sheep and Goats.” Domestic Animal Endocrinology 43: 85–94. 10.1016/j.domaniend.2012.03.005. 22533940

[jez70101-bib-0021] Ibidhi, R. , A. Frija , M. Jaouad , and H. Ben Salem . 2018. “Typology Analysis of Sheep Production, Feeding Systems and Farmers Strategies for Livestock Watering in Tunisia.” Small Ruminant Research 160: 44–53. 10.1016/j.smallrumres.2018.01.010.

[jez70101-bib-0022] Indu, S. , V. Sejian , D. Kumar , A. Pareek , and S. M. K. Naqvi . 2015. “Ideal Proportion of Roughage and Concentrate for Malpura Ewes to Adapt and Reproduce in a Semi‐Arid Tropical Environment.” Tropical Animal Health and Production 47: 1487–1495. 10.1007/s11250-015-0889-1. 26205905

[jez70101-bib-0023] Jarrige, R. 1988. Alimentation des Bovins, Ovins et Caprins. INRA Editions.

[jez70101-bib-0024] Khaldi, G. 1984. “Variations Saisonnières de l'Activité Ovarienne, du Comportement d'Oestrus et de la Durée de l'Anoestrus Post‐partum des Femelles Ovines de Race Barbarine: Influence du Niveau Alimentaire et de la Présence du Mâle.” Thèse Doct. Etat Sci., Université de Montpellier.

[jez70101-bib-0025] Khaldi, G. , and M. Farid . 1981. “Encyclopédie des Productions Animales dans le Monde Arabe.” In La Tunisie, 214. ACSAD.

[jez70101-bib-0026] Kleemann, D. O. , and S. K. Walker . 2005. “Fertility in South Australian Commercial Merino Flocks: Relationships Between Reproductive Traits and Environmental Cues.” Theriogenology 63: 2416–2433. 10.1016/j.theriogenology.2004.09.052. 15910923

[jez70101-bib-0027] Kumar, D. , K. De , and V. Sejian . 2017. “Impact of Climate Change on Sheep Reproduction.” In Sheep Production Adapting to Climate Change, edited by V. Seijan , R. Bhatta , J. Gaughan , P. Malik , S. Naqvi and R. Lal , 71–93. 10.1007/978-981-10-4714-5_3.

[jez70101-bib-0028] Lassoued, N. , and G. Khaldi . 1995. “Variations saisonnières de l'activité sexuelle des brebis de races Queue Fine de l'Ouest et Noire de Thibar.” In L'Elevage Ovin en Zones Arides et Semi‐arides, 27–34. CIHEAM.

[jez70101-bib-0029] Lassoued, N. , M. Rekik , F. Maatoufi , and I. Ben Salem . 2008. “Summer Solar Radiation and Reproductive Performances in Barbarine Sheep Raised in Semi arid Conditions.” In Proceedings of the International Conference on Livestock and Global Climate Change, edited by P. Rowlinson , M. Steele and A. Nefzaoui , 201. British Society of Animal Science.

[jez70101-bib-0030] Macías‐Cruz, U. , A. Correa‐Calderón , M. Mellado , C. A. Meza‐Herrera , C. F. Aréchiga , and L. Avendaño‐Reyes . 2018. “Thermoregulatory Response to Outdoor Heat Stress of Hair Sheep Females at Different Physiological State.” International Journal of Biometeorology 62: 2151–2160. 10.1007/s00484-018-1615-2. 30244321

[jez70101-bib-0031] Macías‐Cruz, U. , M. A. Gastélum , and F. D. Álvarez , et al. 2016. “Effects of Summer Heat Stress on Physiological Variables, Ovulation and Progesterone Secretion in Pelibuey Ewes Under Natural Outdoor Conditions in an Arid Region.” Animal Science Journal 87: 354–360. 10.1111/asj.12430. 26304696

[jez70101-bib-0032] Marai, I. F. M. , A. A. E.‐ Darawany , A. Fadiel , and M. A. M. Abdel‐Hafez . 2008. “Reproductive Performance Traits as Affected by Heat Stress and its Alleviation in Sheep.” Tropical and Subtropical Agroecosystems 8: 209–234.

[jez70101-bib-0033] Marai, I. F. M. , A. A. El‐Darawany , A. Fadiel , and M. A. M. Abdel‐Hafez . 2007. “Physiological Traits as Affected by Heat Stress in Sheep—A Review.” Small Ruminant Research 71: 1–12. 10.1016/j.smallrumres.2006.10.003.

[jez70101-bib-0034] McManus, C. , G. R. Paludo , H. Louvandini , R. Gugel , L. C. B. Sasaki , and S. R. Paiva . 2009. “Heat Tolerance in Brazilian Sheep: Physiological and Blood Parameters.” Tropical Animal Health and Production 41: 95–101. 10.1007/s11250-008-9162-1. 19052907

[jez70101-bib-0035] McManus, C. M. , D. A. Faria , C. M. Lucci , H. Louvandini , S. A. Pereira , and S. R. Paiva . 2020. “Heat Stress Effects on Sheep: Are Hair Sheep More Heat Resistant?” Theriogenology 155: 157–167. 10.1016/j.theriogenology.2020.05.047. 32679441

[jez70101-bib-0036] Mehaba, N. , A. A. K. Salama , X. Such , E. Albanell , and G. Caja . 2019. “Lactational Responses of Heat‐Stressed Dairy Goats to Dietary L‐Carnitine Supplementation.” Animals 9: 567. 10.3390/ani9080567. 31426431 PMC6718979

[jez70101-bib-0037] Mleil, S. , N. Lassoued , H. B. Salem , and K. Kraïem , 2011. Effect of Water Deprivation During Last Pregnancy and Post‐Partum Period on Barbarine Ewes Performances and Lambs' Growth.” In Challenging Strategies to Promote the Sheep and Goat Sector in the Current Global Context., edited by M. J. Ranilla , M. D. Carro , H. S. Ben and P. Morand‐Fehr , 279–283. CIHEAM.

[jez70101-bib-0038] Mohamed‐Brahmi, A. , M. Ameur , and I. Mekki , et al. 2024. “Analysis of Management Practices and Breeders’ Perceptions of Climate Change's Impact to Enhance the Resilience of Sheep Production Systems: A Case Study in the Tunisian Semi‐Arid Zone.” Animals 14: 885. 10.3390/ani14060885. 38539982 PMC10967291

[jez70101-bib-0039] Moradi, M. H. , A. Nejati‐Javaremi , M. Moradi‐Shahrbabak , K. G. Dodds , and J. C. McEwan . 2012. “Genomic Scan of Selective Sweeps in Thin and Fat Tail Sheep Breeds for Identifying of Candidate Regions Associated With Fat Deposition.” BMC Genetics 13: 10. 10.1186/1471-2156-13-10. 22364287 PMC3351017

[jez70101-bib-0040] Ben Moula, A. , A. Kchikich , M. Chentouf , et al. 2024. “Climate Change Impacts on Sheep and Goat Production and Reproduction.” Journal of Central European Agriculture 25: 910–918. 10.5513/JCEA01/25.4.4335.

[jez70101-bib-0041] Naqvi, S. M. K. , V. P. Maurya , R. Gulyani , A. Joshi , and J. P. Mittal . 2004. “The Effect of Thermal Stress on Superovulatory Response and Embryo Production in Bharat Merino Ewes.” Small Ruminant Research 55: 57–63. 10.1016/j.smallrumres.2004.02.009.

[jez70101-bib-0042] Ngcobo, J. N. , I. Egerszegi , and K. A. Nephawe . 2025. “Recent Advances in Understanding the Impact of Environmental Heat Stress on Sheep Production and Reproductive Performance: A Subtropical Climate Perspective.” Climate 13: 130. 10.3390/cli13060130.

[jez70101-bib-0043] OEP . 2020. “Office de l'Elevage et des Pâturages.” Ministère de l'Agriculture, des ressources hydriques et de la pêche maritime. http://www.oep.nat.tn/index.php/fr/donnees-sectorielles/40-effectif-s-du-cheptel.

[jez70101-bib-0044] Omidi, A. , M. Kheirie , and H. Sarir . 2015. “Impact of Vitamin C on Concentrations of Thyroid Stimulating Hormone and Thyroid Hormones in Lambs Under Short‐Term Acute Heat Stress.” Veterinary Science Development 5: 5965. 10.4081/vsd.2015.5965.

[jez70101-bib-0045] Papanastasiou, D. , T. Bartzanas , P. Panagakis , and C. Kittas . 2014. “Assessment of a Typical Greek Sheep Barn Based on Potential Heat‐Stress of Dairy Ewes.” Applied engineering in agriculture 30: 953–959. 10.13031/aea.30.10256.

[jez70101-bib-0046] Papanastasiou, D. K. , P. Panagakis , and V. Anestis , et al. 2018. “Environmental Conditions, Potential Heat‐Stress State and Their Relations in a Sheep Barn Under Hot Climate.” CIGR Journal: 1–13.

[jez70101-bib-0047] Pourlis, A. F. 2011. “A Review of Morphological Characteristics Relating to the Production and Reproduction of Fat‐Tailed Sheep Breeds.” Tropical Animal Health and Production 43: 1267–1287. 10.1007/s11250-011-9853-x. 21556961

[jez70101-bib-0048] Prathap, P. , V. Sejian , and N. M. Soren , et al. 2017. “Summer Season Induced Rhythmic Alterations in Metabolic Activities to Adapt to Heat Stress in Three Indigenous (Osmanabadi, Malabari and Salem Black) Goat Breeds.” Biological Rhythm Research 49: 551–565. 10.1080/09291016.2017.1386891.

[jez70101-bib-0049] Rathwa, S. D. , A. A. Vasava , M. M. Pathan , S. P. Madhira , Y. G. Patel , and A. M. Pande . 2017. “Effect of Season on Physiological, Biochemical, Hormonal, and Oxidative Stress Parameters of Indigenous Sheep.” Veterinary World 10: 650–654. 10.14202/vetworld.2017.650-654. 28717317 PMC5499082

[jez70101-bib-0050] Reece, W. O. , and E. W. Rowe . 2017. “Body heat and temperature regulation.” In Functional Anatomy and Physiology of Domestic Animals, edited by W. O. Reece and E. W. Rowe , 5th edn., 402–411. Wiley Blackwell.

[jez70101-bib-0051] Rekik, M. , R. Aloulou , and M. Ben Hamouda . 2005. “Small Ruminant Breeds of Tunisia.” Characterisation of Small Ruminant Breeds in West Asia and North Africa 2: 91–140.

[jez70101-bib-0052] Renaudeau, D. , A. Collin , S. Yahav , V. de Basilio , J. L. Gourdine , and R. J. Collier . 2012. “Adaptation to Hot Climate and Strategies to Alleviate Heat Stress in Livestock Production.” Animal 6: 707–728. 10.1017/S1751731111002448. 22558920

[jez70101-bib-0053] Rensis, F. D. , and R. J. Scaramuzzi . 2003. “Heat Stress and Seasonal Effects on Reproduction in the Dairy Cow–A Review.” Theriogenology 60: 1139–1151. 10.1016/s0093-691x(03)00126-2. 12935853

[jez70101-bib-0054] Robertson, S. , and M. Friend . 2022. “Strategies to Ameliorate Heat Stress Effects on Sheep Reproduction.” In Climate Change and Livestock Production, edited by V. Sejian , S. S. Chauhan , C. Devaraj , P. K. Malik , and R. Bhatta , 175–183. Springer. 10.1007/978-981-16-9836-1_15.

[jez70101-bib-0055] Rodrigues, R. C. M. , D. A. Furtado , and N. L. Ribeiro , et al. 2023. “Blood Biochemical, Hormonal, and Hematological Responses of Native Sheep Submitted to Different Environmental Conditions.” Research in Veterinary Science 165: 105067. 10.1016/j.rvsc.2023.105067. 37925818

[jez70101-bib-0057] Rojas‐Downing, M. M. , A. P. Nejadhashemi , T. Harrigan , and S. A. Woznicki . 2017. “Climate Change and Livestock: Impacts, Adaptation, and Mitigation.” Climate Risk Management 16: 145–163. 10.1016/j.crm.2017.02.001.

[jez70101-bib-0058] Russel, A. J. F. , J. M. Doney , and R. G. Gunn . 1969. “Subjective Assessment of Body Fat in Live Sheep.” Journal of Agricultural Science 72: 451–454. 10.1017/S0021859600024874.

[jez70101-bib-0059] Ben Salem, H. , N. Lassoued , and M. Rekik . 2011. “Merits of the Fat‐Tailed Barbarine Sheep Raised in Different Production Systems in Tunisia: Digestive, Productive and Reproductive Characteristics.” Tropical Animal Health and Production 43: 1357–1370. 10.1007/s11250-011-9863-8. 21533615

[jez70101-bib-0060] Santos, M. L. P. , J. M. V. Dada , P. C. Muniz , M. L. A. Nunes‐Zotti , F. R. O. Barros , and F. M. C. Vieira . 2021. “Physiological Responses of Santa Inês x Dorper Ewes and Lambs to Thermal Environment of Silvopasture and Open Pasture Systems.” Small Ruminant Research 205: 106565. 10.1016/j.smallrumres.2021.106565.

[jez70101-bib-0061] SAS Institute Inc . 2017. SAS® 9.4 Software. SAS Institute Inc.

[jez70101-bib-0062] Sejian, V. , M. Bagath , G. Krishnan , et al. 2019. “Genes for Resilience to Heat Stress in Small Ruminants: A Review.” Small Ruminant Research 173: 42–53. 10.1016/j.smallrumres.2019.02.009.

[jez70101-bib-0063] Sejian, V. , R. Bhatta , J. Gaughan , P. K. Malik , S. M. K. Naqvi , and R. Lal . 2017. “Adapting Sheep Production to Climate Change.” In Sheep Production Adapting to Climate Change, edited by V. Sejian , R. Bhatta , J. Gaughan , P. K. Malik , S. M. K. Naqvi , and R. Lal , 1–29. Springer Singapore. 10.1007/978-981-10-4714-5_1.

[jez70101-bib-0064] Sejian, V. , R. Bhatta , J. B. Gaughan , F. R. Dunshea , and N. Lacetera . 2018. “Review: Adaptation of Animals to Heat Stress.” Animal 12: s431–s444. 10.1017/S1751731118001945. 30139399

[jez70101-bib-0065] Sejian, V. , V. P. Maurya , K. Kumar , and S. M. K. Naqvi . 2012. “Effect of Multiple Stresses (Thermal, Nutritional, and Walking Stress) on the Reproductive Performance of Malpura Ewes.” Veterinary Medicine International 2012: 471760. 10.1155/2012/471760. 22448337 PMC3289860

[jez70101-bib-0066] Silanikove, N. 2000. “Effects of Heat Stress on the Welfare of Extensively Managed Domestic Ruminants.” Livestock Production Science 67: 1–18. 10.1016/S0301-6226(00)00162-7.

[jez70101-bib-0067] Thimonier, J. , P. Mauléon , J. Bézard , M.‐M. D. Reviers , and C. Cornu . 1969. “Variations Saisonnières du Comportement d’œstrus et des Activités Ovarienne et Hypophysaire Chez les Ovins.” Annales de Biologie Animale, Biochimie, Biophysique 9: 233–250. 10.1051/rnd:19690207.

[jez70101-bib-0068] Todaro, M. , M. Dattena , A. Acciaioli , et al. 2015. “Aseasonal Sheep and Goat Milk Production in the Mediterranean Area: Physiological and Technical Insights.” Small Ruminant Research 126: 59–66. 10.1016/j.smallrumres.2015.01.022.

[jez70101-bib-0069] Todini, L. 2007. “Thyroid Hormones in Small Ruminants: Effects of Endogenous, Environmental and Nutritional Factors.” Animal 1: 997–1008. 10.1017/S1751731107000262. 22444802

[jez70101-bib-0070] Tüfekci, H. 2023. “The Effects of Heat Stress on Some Parameters in Sheep and Goats.” Conference: 11th International Conference on Agriculture, Animal Science and Rural Development.

[jez70101-bib-0075] United States Agency for International Development (USAID) . 2018. Climate Risk Profile: Tunisia. USAID.

[jez70101-bib-0071] Vieira, F. M. C. , J. A. Pilatti , and Z. M. W. Czekoski , et al. 2021. “Effect of the Silvopastoral System on the Thermal Comfort of Lambs in a Subtropical Climate: A Preliminary Study.” Agriculture 11: 790. 10.3390/agriculture11080790.

[jez70101-bib-0072] van Wettere, W. H. E. J. , K. L. Kind , K. L. Gatford , et al. 2021. “Review of the Impact of Heat Stress on Reproductive Performance of Sheep.” Journal of Animal Science and Biotechnology 12: 26. 10.1186/s40104-020-00537-z. 33583422 PMC7883430

[jez70101-bib-0073] Yuan, J.‐D. , L.‐W. Wang , and S.‐Y. Fu , et al. 2025. “Heat Tolerance Differences Between Hu Sheep and Hu Crossbred Sheep in Microbial Community Structure and Metabolism.” Metabolites 15, no. 1: 40. 10.3390/metabo15010040. 39852383 PMC11768064

[jez70101-bib-0074] Čukić, A. , S. Rakonjac , and R. Djoković , et al. 2023. “Influence of Heat Stress on Body Temperatures Measured by Infrared Thermography, Blood Metabolic Parameters and Its Correlation in Sheep.” Metabolites 13: 957. 10.3390/metabo13080957. 37623900 PMC10456689

